# COVID-19-related information sources and anxiety levels

**DOI:** 10.1192/j.eurpsy.2022.785

**Published:** 2022-09-01

**Authors:** T. Ionescu, C. Tudose

**Affiliations:** University of Medicine and Pharmacy Carol Davila, Neuroscience 6, Bucharest, Romania

**Keywords:** Covid-19, Anxiety, information sources

## Abstract

**Introduction:**

Timely and accurate information is foundational to moderating and curing the COVID-19 for both the public and the scientific community, while repeated media exposure to crisis-related information raises stress and anxiety among general population.

**Objectives:**

The main goal of the current study was to evaluate the associations between the COVID-19-related information sources and anxiety levels.

**Methods:**

An online web-survey recruited participants who were ≥18 years old and lived in Romanian through an online campaign in May 2021. Participants were asked whether they received COVID-19-related information frequently from the following sources: the Internet, traditional media, medical staff in health care settings or from friends, co-workers, or family members. We also assessed participants’ level of anxiety with Zung Self-Rating Anxiety Scale (SAS) and the cut-off point for anxiety index was set at 45. The associations of each information source with anxiety were examined using multiple regression analyses to control for sex, age, education and other demographic characteristics.

**Results:**

In total, the data of 1559 respondents (1224 female; mean age = 37.03 years and standard deviation (SD)=12.90 years) were analysed. The mean index score for SAS were 44.28 (SD=10.6). The major source of information on COVID-19 was the Internet (59.20%) and medical stuff (58.27%), almost in equal measure, followed by traditional media (48.17%) and friends, co-workers, and family members (30.72%).

**Conclusions:**

Receiving COVID-19 information from the Internet and traditional media was positively correlated with anxiety level (p=0.01), while receiving COVID-19 information from medical-stuff was associated with low levels of anxiety (p=0.03).

**Disclosure:**

No significant relationships.

